# Supplementary material for: Structural insights into Legionella RidL-Vps29 retromer subunit interaction reveal displacement of the regulator TBC1D5
Source: Nat Commun. 2017 Nov 16;8:1543. doi: 10.1038/s41467-017-01512-5 (PMC5691146; doi:10.1038/s41467-017-01512-5)
Supplement: Supplementary file 1 — Supplementary Information [file 41467_2017_1512_MOESM1_ESM.pdf]

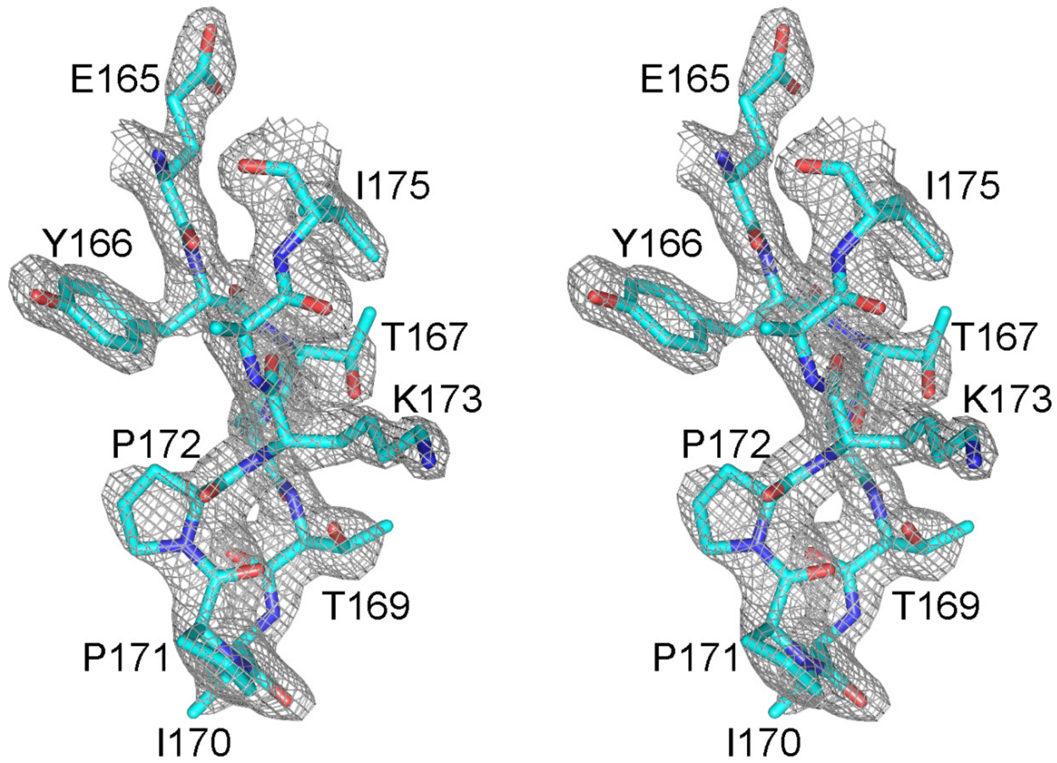

Supplementary Fig. 1. Stereo view of the β-hairpin. The 2Fo-Fc electron density map is contoured at 1.5 σ.

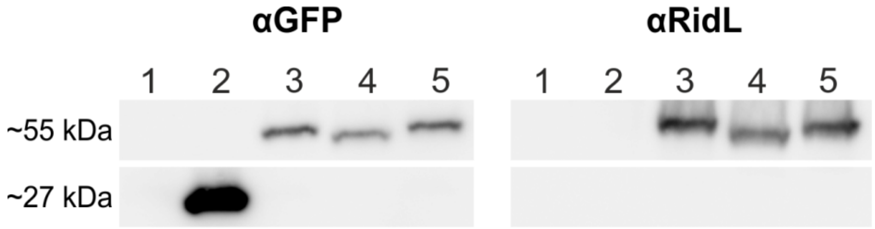

Supplementary Fig. 2. Production of GFP fusion proteins by transfected HeLa cells. HeLa cells were transiently transfected with the GFP fusion proteins or GFP as indicated. After 24 h, the transfected cells were detached and lysed. Total cell extracts were separated by SDS-PAGE, blotted and immuno-stained for GFP (left panels) or RidL (right panels), respectively. Lane 1, no plasmid; lane 2, GFP (26.9 kDa), lane 3, RidL$_{9-258}$-GFP (55.9 kDa), lane 4, RidL$_{9-258}$-Δβ-hairpin-GFP (53.9 kDa); lane 5, RidL$_{9-258}$-I$_{170}$E-GFP (56.0 kDa).

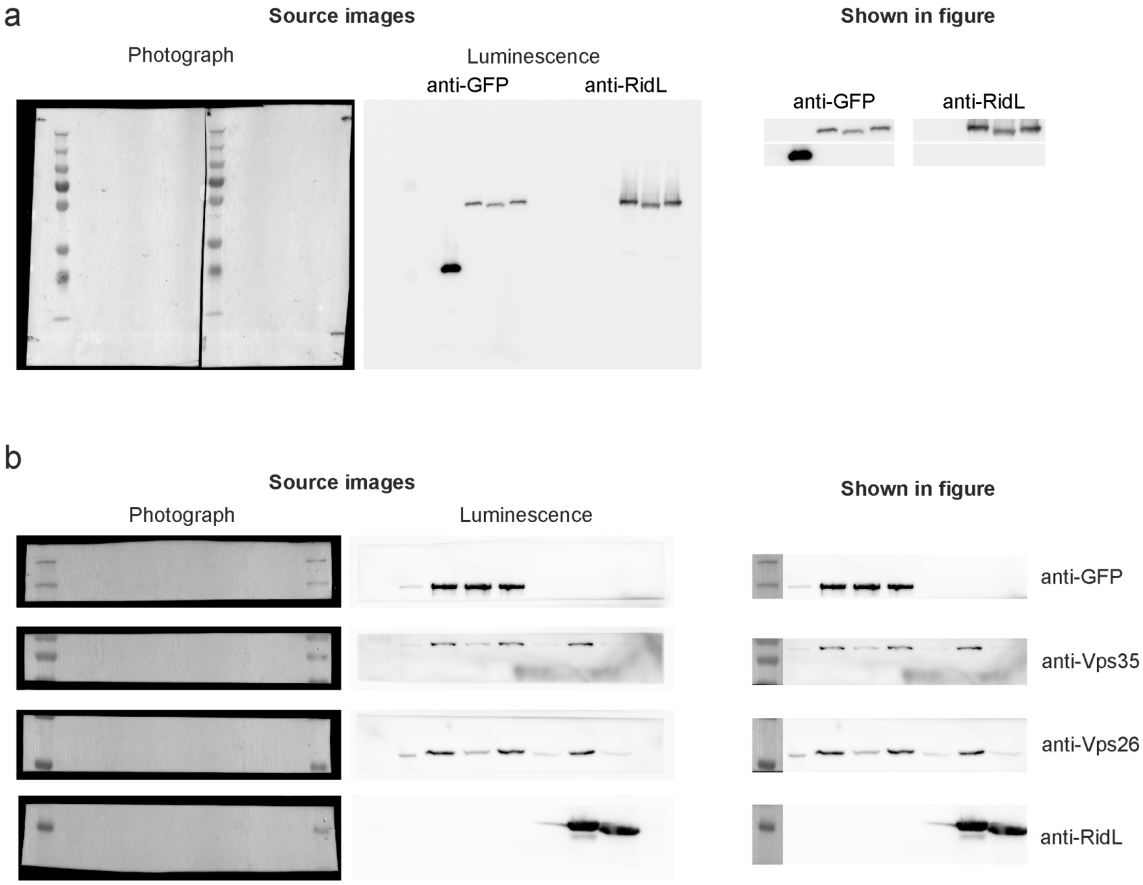

Supplementary Fig. 3. Western blot source data. Source data for Western blots shown in (**a**) Supplementary Fig. 2 and (**b**) Fig. 5c. The nitrocellulose membrane shown in (b) was cut horizontally to allow exposure of an identical sample to different antibodies in parallel.

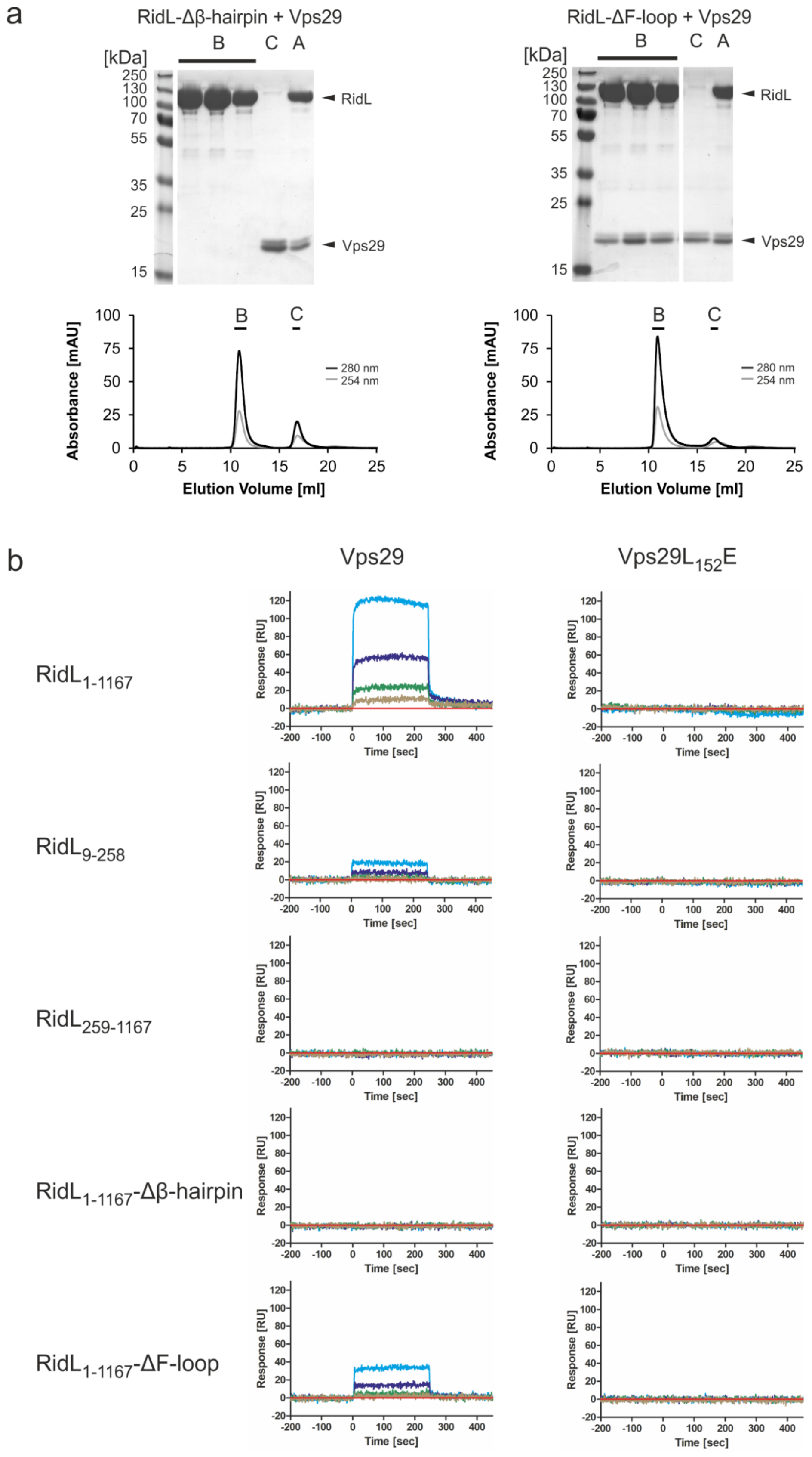
a
RidL-Δβ-hairpin + Vps29
RidL-ΔF-loop + Vps29
[kDa]
B
C
A
RidL
Vps29
250
130
100
70
55
35
25
15
Absorbance [mAU]
Elution Volume [ml]
280 nm
254 nm
b
Vps29
Vps29L152E
RidL1-1167
RidL9-258
RidL259-1167
RidL1-1167-Δβ-hairpin
RidL1-1167-ΔF-loop
Response [RU]
Time [sec]

Supplementary Fig. 4. The RidL retromer-interacting fragment binds Vps29 but not Vps29$_{L152E}$ *in vitro*. (**a**) For size exclusion chromatography (SEC) RidL-Δβ-hairpin or RidL-ΔF-loop deletion mutants (130 kDa) were mixed in a molar ratio 1:1.5 with Vps29 (21 kDa) or Vps29$_{L152E}$ (21 kDa). SDS-PAGE and Coomassie Brilliant Blue staining of A) input (ratio 1:1.5), B) SEC elution fractions 10.5-11.5 ml (elution volume RidL or complex), and C) SEC elution fraction 16.5 ml (elution volume Vps29) were analyzed. SEC elution profiles are shown and analyzed fractions are labelled. (**b**) For surface plasmon resonance (SPR) experiments, biotinylated Vps29 or Vps29$_{L152E}$ was used as ligand, and different RidL variants were used as analytes. Concentration series of analytes (red: 0 nM, brown: 50 nM, green: 150 nM, blue: 450 nM, cyan: 1.35 μM) were added from time point 0 to 250 sec. Response in arbitrary units (RU) are plotted against time (sec). Association and dissociation of ligands to analytes were too fast to be quantified, the binding equilibrium signals did not saturate within the measured range of analyte concentrations, and thus, the SPR sensograms were interpreted qualitatively.

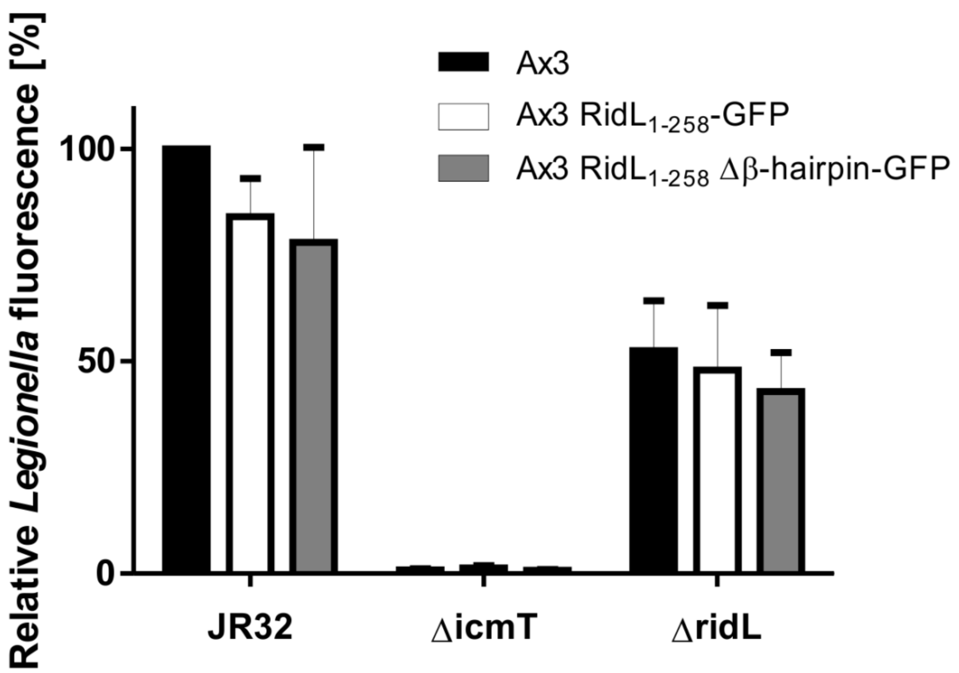

Supplementary Fig. 5. Ectopic production of RidL N-terminal domain does not affect intracellular replication of *L. pneumophila*. *D. discoideum* Ax3 producing RidL$_{1\text{-}258}$-GFP, RidL$_{1\text{-}258}$-Δβ-hairpin-GFP or no RidL fragment was infected (MOI 1, 6 d) with *L. pneumophila* JR32, Δ*ridL* or Δ*icmT* mutant bacteria harboring plasmid pNT-28, and intracellular replication was determined by fluorescence. Background fluorescence of uninfected amoebae was subtracted. Mean of fluorescence relative to JR32 in Ax3 and SD of three independent experiments (each in technical triplicates) is shown.

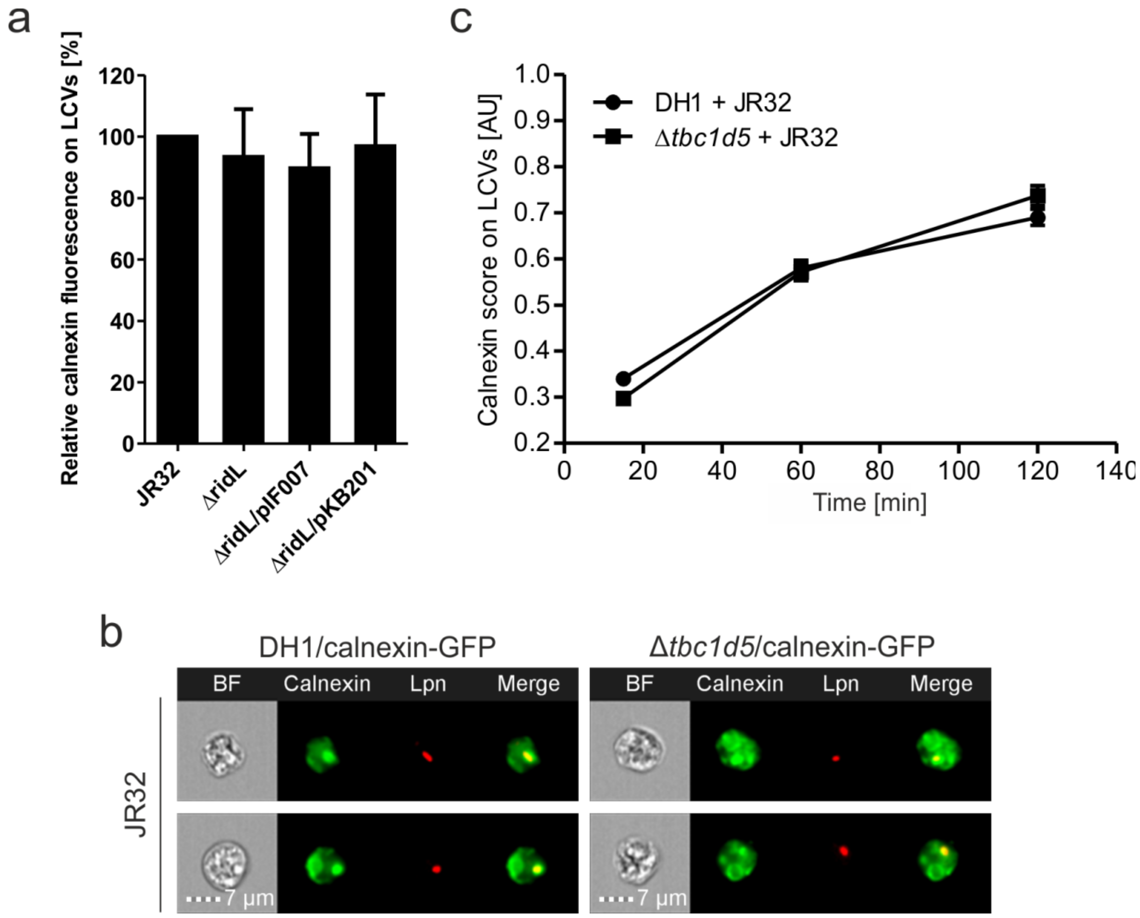

Supplementary Fig. 6. RidL or TBC1D5 do not affect recruitment of calnexin to LCVs. (**a**) *D. discoideum* DH1 stably producing GFP-TBC1D5 were infected (MOI 20, 2 h) with DsRed-producing virulent *L. pneumophila* JR32 or Δ*ridL* harboring pCR77 (vector), Δ*ridL*/pIF007 (RidL) or Δ*ridL*/pKB201 (RidL-Δβ-hairpin). Intact LCVs were purified, immuno-stained for calnexin and analyzed by fluorescence microscopy. Mean and SD of three independent biological replicates (LCV isolations) is shown, each with at least 44 LCVs (average pixel GFP intensity of masked LCVs relative to the value of strain JR32; one way ANOVA). (**b**) Imaging flow cytometry (IFC) images of *D. discoideum* DH1 or Δ*tbc1d5* producing calnexin-GFP (pAW16), infected (MOI 5, 2 h) with mPlum-producing *L. pneumophila* JR32 (pAW14). (**c**) Quantification of IFC colocalization score between GFP and mPlum in >1800 cells per sample at the time points p.i. indicated. Data show mean and 95% confidence intervals of one representative experiment out of three independent biological replicates.

Supplementary Table 1. Strains and plasmids used in this study.

**Strain/plasmid**	**Relevant properties** [a]	**Reference**
D. discoideum		
Ax3	Parental strain	Laboratory strain, [1]
DH1-10	Parental strain	[2]
DH1_ΔTBC1D5	Insertion in gene DDB_G0280253	This work
E. coli		
TOP10		Invitrogen
MC1061	*araD139 Δ(araA-leu)7697 Δ(lac)X74 galK16 galE15(GalS) λ- e14- mcrA0 relA1 rpsL150(strR) spoT1 mcrB1 hsdR2*	[3]
L. pneumophila		
CR06 (Δ*ridL*)	*L. pneumophila* JR32 *ridL*::Kan^R	[1]
GS3011 (Δ*icmT*)	*L. pneumophila* JR32 *icmT3011*::Kan^R	[4]
JR32	Virulent *L. pneumophila* sg 1 strain Philadelphia	[5]
**Plasmids**		
mPlum-pBAD		Addgene, #54564
AmtA-mCherry	pDM1044-*amtA-mCherry*, Hyg^R	[6]
pAW9	pDM317-*gfp-rab7*	This work
pAW14	pMMB207-C, Δ*lacI*^{q} (const. *mPlum*)	This work
pAW16	pDM323-*cnxA-gfp*	This work
pBXC3GH	C-terminal 3C protease cleavable GFP-His10- tag, PBAD, Amp^R	[7]
pBXCA3GH	C-terminal Avi- and 3C protease cleavable GFP-His10- tag, PBAD, Amp^R	[7]
pBXNH3	N-terminal 3C protease cleavable His10-tag, PBAD, Amp^R	[7]
pCaln-GFP	P_{act15}, calnexinA-RSSSKLK-GFP (S65T), G418^R	[8]

pCR77	pMMB207-C-P_{tac}-RBS-*dsred*-RBS-MCS	[1]
pCR90	pGEX-6P-*ridL*	[1]
pCR94	peGFP-C1-*ridL*	[1]
pDM317	*Dictyostelium* expression vector, extra-chromosomal, N-terminal GFP, $G418^R$	[9]
pDM323	*Dictyostelium* expression vector, extra-chromosomal, C-terminal GFP, $G418^R$	[9]
pEF-Bos-Flag-Vps29	Flag-tagged Vps29	[10]
pFL1304	*Dictyostelium* expression plasmid for GFP-TBC1D5	This work
peGFP-C1	Mammalian expression vector, P_{CMV}, Neo^R, Kan^R	Clontech
peGFP-C1-TBC1D5	encoding GFP-TBC1D5	[11]
peGFP-N1	Mammalian expression vector, P_{CMV}, Neo^R, Kan^R	Clontech
pIF007	pMMB207-C-P_{tac}-RBS-*dsred*-RBS-MCS-*ridL*	[1]
pINIT	Cloning plasmid, Cam^R	[7]
pKB002	pINIT-*ridL*	This work
pKB004	pINIT-*vps29*	This work
pKB016	pBXC3GH-*ridL*	This work
pKB024	pBXC3GH-*vps29*	This work
pKB044	pINIT-$vps29_{L152E}$	This work
pKB045	pBXC3GH-$vps29_{L152E}$	This work
pKB108	pBXCA3GH-*ridL*	This work
pKB109	pBXCA3GH-*vps29*	This work
pKB128	pINIT-$ridL_{2-280}$	This work
pKB134	pBXNH3-$ridL_{2-281}$	This work
pKB136	pINIT-$ridL_{10-258}$	This work
pKB137	pBXNH3-$ridL_{10-258}$	This work
pKB144	pBXCA3GH-$vps29_{L152E}$	This work
pKB149	peGFP-C1-*ridL-Δβ-hairpin*	This work
pKB150	peGFP-C1-*ridL-ΔF-loop*	This work

pKB151	pINIT-*ridL*-Δ*β-hairpin*	This work
pKB152	pINIT-*ridL*-Δ*F-loop*	This work
pKB153	pBXC3GH-*ridL*-Δ*β-hairpin*	This work
pKB154	pBXC3GH-*ridL*-Δ*F-loop*	This work
pKB158	pINIT-$ridL_{10\text{-}258}$-Δ*β-hairpin*	This work
pKB161	pBXNH3-$ridL_{10\text{-}258}$-Δ*β-hairpin*	This work
pKB164	pINIT-$ridL_{259\text{-}1167}$	This work
pKB167	pBXC3GH-$ridL_{259\text{-}1167}$	This work
pKB185	peGFP-N1-$ridL_{9\text{-}258}$	This work
pKB201	pMMB207-C-P_{tac}-RBS-*dsred*-RBS-*ridL*-Δ*β-hairpin*	This work
pKB203	pDM323-$ridL_{1\text{-}258}$-*gfp*	This work
pKB216	peGFP-N1-*ridL*-Δ*β-hairpin*	This work
pKB234	peGFP-N1-$ridL_{9\text{-}258}$-Δ*β-hairpin*	This work
pKB235	pINIT-$ridL_{I170E}$	This work
pKB236	peGFP-N1-$ridL_{9\text{-}258}$-$_{I170E}$	This work
pKB269	pDM323-$ridL_{1\text{-}258}$-Δ*β-hairpin-gfp*	This work
pNT28	pMMB207-C, Δ$lacI^{q}$ (constitutive *gfp*)	[12]
pSU23	pDXA-HC-eGFP-*rab7*	[13]
pWS21	Calnexin-mRFPmars, Bls^{R}	[14]

[a] Abbreviations: Amp, ampicillin; Bls, blasticidin S; Cam, chloramphenicol; Hyg, hygromycin; Kan, kanamycin; Neo, neomycin; G418, geneticin.

Supplementary Table 2. Oligonucleotides used in this study.

Oligo	Sequence (5' - 3') [a]	Comments
oAW13	ATATATGGATCCATGGCCACAAAGAAAAAG	5' of *rab7*, BamHI
oAW14	ATATATACTAGTACAACAACCTGATTTAGCTG	3' of *rab7*, SpeI
oAW30_fwd	ATATATGAATTC**GCTAGATTTAAGAAGGAGATATACAT** ATGGTG AGCAAGGGCGAGGAG	5' of *mPlum*, EcoRI, RBS bold
oAW31_rev	ATATATAAGCTTTTAG GCGCCGGTGGAGTGGC	3' of *mPlum*, HindIII
oAW35	ATATATGGATCCATGAATAAATTAATTTATTATTAATTT TATC	5' of *cnxA*, BamHI
oAW36	ATATATACTAGTTTTAACTTTATTAGTTCTTTTTG	3' of *cnxA*, SpeI
oCR126	AAAAACGCGTCGACATGATTCTCGAGGAGTACATCC	5' of *ridL*, peGFP-N1, SalI
oKB001	AGCTGCTCTTCTAGTATTCTCGAGGAGTACATCC	5' of *ridL*, BspQI
oKB002	TATAGCTCTTCCTGCCTTACGCATCCCTGTAC	3' of *ridL*, BspQI
oKB003	CTGTGCTCTTCTAGTTTGGTGTTGGTATTAGGAG	5' of *vps29*, BspQI
oKB004	CAGAGCTCTTCATGCAGGTTTCTTGTATTCGATTCG	3' of *vps29*, BspQI
oKB038	CATTTGTGGAGATGGATATCCAGGCTTCTAC	SDM, $vps29_{L152E}$
oKB039	GATATCCATCTCCACAAATGATGGAATAATG	SDM, $vps29_{L152E}$
oKB076	ATATATGCTCTTCTAGTGCCAAAAATAAAGAATTTTTTG	5' of $ridL_9$, BspQI
oKB080	TATATAGCTCTTCATGCTAATAGCGCATTGTGAGATAAT	3' of $ridL_{280}$, BspQI
oKB082	TATATAGCTCTTCATGCTAGTTTTCAACCCTTTCTTCC	3' of $ridL_{258}$, BspQI
oKB084	TGCATGGTGGCAGCGGCGGTGTTTTATCTGATGAAGCG	SDM, Δβ-hairpin, 3' fragment
oKB085	TAAAACACCGCCGCTGCCACCATGCACATTTCCTTCAC	SDM, Δβ-hairpin, 5' fragment
oKB088	TCTGCAGTCGACATGATTC	SDM loop mutants, 5' fragment
oKB089	GCTCATTAATTTCGAATGGGC	SDM loop mutants, 3' fragment

oKB090	GGAGGGTGGTAGCGGCTTTTTAAATTCTACAGATCCAAC	SDM, ΔF-loop, 5’ fragment
oKB091	ATTTAAAAAGCCGCTACCACCCTCCATCAAGCTAGGATC	SDM, ΔF-loop, 3’ fragment
oKB095	ATTTAGTCGACATGGCCAAAAATAAAGAATTTTTTG	5’ of $ridL_{9}$, peGFP-N1, SalI
oKB098	ATATATGCTCTTCTAGTGAACAGGAAGCTGCCAAG	5’ of $ridL_{259}$, BspQI
oKB116	ATTTAGGATCCCCTAGTTTTTCAACCCTTTCTTCC	3’ of $ridL_{258}$, peGFP-N1, BamHI
oKB127	ATTTAAGATCTAAAATGATTCTCGAGGAGTACATCCG	5’ of *ridL*, pDM323, BglII
oKB130	ATTTAACTAGTTAGTTTTTCAACCCTTTCTTCCAG	3’ of $ridL_{258}$, pDM323, BcuI
oKB132	ATTTAGGATCCCCCTTACGCATCCCTGTACTCG	3’ of *ridL*, peGFP-N1, BamHI
oKB136	CCGACAGAACCTCCGAAAGCTATTAAC	SDM, $ridL_{I170E}$
oKB137	CGGAGGTTCTGTCGGGGTATATTCTTC	SDM, $ridL_{I170E}$

[a] Nucleotides mutated by site-directed mutagenesis (SDM) are underlined.

Supplementary References

1. Finsel I, Ragaz C, Hoffmann C, Harrison CF, Weber S, van Rahden VA, Johannes L, Hilbi H (2013) The *Legionella* effector RidL inhibits retrograde trafficking to promote intracellular replication. *Cell Host Microbe* **14:** 38-50
2. Cornillon S, Pech E, Benghezal M, Ravanel K, Gaynor E, Letourneur F, Bruckert F, Cosson P (2000) Phg1p is a nine-transmembrane protein superfamily member involved in *Dictyostelium* adhesion and phagocytosis. *J Biol Chem* **275:** 34287-34292
3. Casadaban MJ, Cohen SN (1980) Analysis of gene control signals by DNA fusion and cloning in *Escherichia coli*. *J Mol Biol* **138:** 179-207
4. Segal G, Shuman HA (1998) Intracellular multiplication and human macrophage killing by *Legionella pneumophila* are inhibited by conjugal components of IncQ plasmid RSF1010. *Mol Microbiol* **30:** 197-208
5. Sadosky AB, Wiater LA, Shuman HA (1993) Identification of *Legionella pneumophila* genes required for growth within and killing of human macrophages. *Infect Immun* **61:** 5361-5373
6. Barisch C, Paschke P, Hagedorn M, Maniak M, Soldati T (2015) Lipid droplet dynamics at early stages of *Mycobacterium marinum* infection in *Dictyostelium*. *Cell Microbiol* **17:** 1332-1349
7. Geertsma ER, Dutzler R (2011) A versatile and efficient high-throughput cloning tool for structural biology. *Biochemistry* **50:** 3272-3278
8. Müller-Taubenberger A, Lupas AN, Li H, Ecke M, Simmeth E, Gerisch G (2001) Calreticulin and calnexin in the endoplasmic reticulum are important for phagocytosis. *EMBO J* **20:** 6772-6782
9. Veltman DM, Akar G, Bosgraaf L, Van Haastert PJM (2009) A new set of small, extrachromosomal expression vectors for *Dictyostelium discoideum*. *Plasmid* **61:** 110-118
10. Rojas R, van Vlijmen T, Mardones GA, Prabhu Y, Rojas AL, Mohammed S, Heck AJ, Raposo G, van der Sluijs P, Bonifacino JS (2008) Regulation of retromer recruitment to endosomes by sequential action of Rab5 and Rab7. *J Cell Biol* **183:** 513-526
11. Seaman MN, Harbour ME, Tattersall D, Read E, Bright N (2009) Membrane recruitment of the cargo-selective retromer subcomplex is catalysed by the small GTPase Rab7 and inhibited by the Rab-GAP TBC1D5. *J Cell Sci* **122:** 2371-2382
12. Tiaden A, Spirig T, Weber SS, Brüggemann H, Bosshard R, Buchrieser C, Hilbi H (2007) The *Legionella pneumophila* response regulator LqsR promotes host cell interactions as an element of the virulence regulatory network controlled by RpoS and LetA. *Cell Microbiol* **9:** 2903-2920
13. Urwyler S, Nyfeler Y, Ragaz C, Lee H, Mueller LN, Aebersold R, Hilbi H (2009) Proteome analysis of *Legionella* vacuoles purified by magnetic immunoseparation reveals secretory and endosomal GTPases. *Traffic* **10:** 76-87
14. Weber S, Wagner M, Hilbi H (2014) Live-cell imaging of phosphoinositide dynamics and membrane architecture during *Legionella* infection. *MBio* **5:** e00839-13
