# Supplementary material for: Structural insights into Legionella RidL-Vps29 retromer subunit interaction reveal displacement of the regulator TBC1D5
Source: Nat Commun. 2017 Nov 16;8:1543. doi: 10.1038/s41467-017-01512-5 (PMC5691146; doi:10.1038/s41467-017-01512-5)
Supplement: Supplementary file 3 — Description of Additional Supplementary Files [file 41467_2017_1512_MOESM3_ESM.pdf]

Description of Supplementary Files

File name: Supplementary Movie 1
Description: Crystal structure of the 29 kDa N-terminal domain of RidL at 1.9 Å resolution, comprising amino acids 2 (dark blue) - 281 (red) of a total of 1167 amino acids. The β-hairpin (aa 160-180) is shown in yellow and the F-loop (aa 75-93) in pale blue.
